# OSA Upper Airways Surgery: A Targeted Approach

**DOI:** 10.3390/medicina57070690

**Published:** 2021-07-06

**Authors:** Andrea De Vito, B. Tucker Woodson, Venkata Koka, Giovanni Cammaroto, Giannicola Iannella, Marcello Bosi, Stefano Pelucchi, Giulio Romano Filograna-Pignatelli, Pierre El Chater, Claudio Vicini

**Affiliations:** 1 Ear Nose Throat (ENT) Unit, Head & Neck Department, Santa Maria delle Croci Hospital, Romagna Health Service, 48121 Ravenna, Italy; giulioromano.filogranapignatelli@auslromagna.it; 2 Division of Sleep Medicine and Upper Airway Reconstructive Surgery, Department of Otolaryngology, Medical College of Wisconsin, Milwaukee, WI 53226, USA; bwoodson@mcw.edu; 3 Department of Sleep Medicine, Hospital Antoine Beclere, 92140 Clamart, France; vkoka@me.com; 4 Ear Nose Throat (ENT) Unit, Head & Neck Department, Morgagni-Pierantoni Hospital, Romagna Health Service, 47121 Forlì, Italy; giovanni.cammaroto@auslromagna.it (G.C.); giannicola.iannella@auslromagna.it (G.I.); claudio.vicini@auslromagna.it (C.V.); 5 Private Hospitals Group, 47121 Forlì, Italy; marcello.bosi@libero.it; 6 Clinic of Otorhinolaryngology, Neuroscience and Rehabilitation Department, University of Ferrara, 44121 Ferrara, Italy; stefano.pelucchi@unife.it; 7 ENT Department, Sulaiman, Al Habib Hospital, Dubai Health Care City, 505005 Dubai, United Arab Emirates; elchaterpierre@gmail.com

**Keywords:** sleep-disordered breathing, apnea, upper airway surgery, continuous positive airway pressure, mandibular advancement device, uvulopharyngopalatoplasty, expansion sphincter palatoplasy, barbed palatoplasty, transoral robotic surgery

## Abstract

Obstructive sleep apnea syndrome (OSA) is a multi-factorial disorder, with quite complex endotypes, consisting of anatomical and non-anatomical pathophysiological factors. Continuous positive airway pressure (CPAP) is recognized as the first-line standard treatment for OSA, whereas upper airway (UA) surgery is often recommended for treating OSA patients who have refused or cannot tolerate CPAP. The main results achievable by the surgery are UA expansion, and/or stabilization, and/or removal of the obstructive tissue to different UA levels. The site and pattern of UA collapse identification is of upmost importance in selecting the customized surgical procedure to perform, as well as the identification of the relation between anatomical and non-anatomical factors in each patient. Medical history, sleep studies, clinical examination, UA endoscopy in awake and drug-induced sedation, and imaging help the otorhinolaryngologist in selecting the surgical candidate, identifying OSA patients with mild UA collapsibility or tissue UA obstruction, which allow achievement of the best surgical outcomes. Literature data reported that the latest palatal surgical procedures, such as expansion sphincter palatoplasty or barbed reposition palatoplasty, which achieve soft palatal and lateral pharyngeal wall remodeling and stiffening, improved the Apnea Hypopnea Index, but the outcome analyses are still limited by methodological bias and the limited number of patients’ in each study. Otherwise, the latest literature data have also demonstrated the role of UA surgery in the improvement of non-anatomical factors, confirming that a multidisciplinary and multimodality diagnostic and therapeutical approach to OSA patients could allow the best selection of customized treatment options and outcomes.

## 1. Introduction

Obstructive sleep apnoea (OSA) represents the most common and under-diagnosed sleep-disordered breathing (SDB) disease, with an incidence rate between 5 and 17% of the middle-aged population and 20 to 60% in people over 65 [1,2].

Currently, the pathophysiology of OSA is related to four major endotypes related to specific pathophysiological traits (PTs): the upper airway size/pharyngeal collapsibility, the upper airway muscular responsiveness, the ventilatory control system or loop gain (LG), and the arousal threshold (AT). Even though the upper airway size/pharyngeal collapsibility is the most important factor amongst the PTs, the relative contribution or combination of each of the PTs to OSA varies substantially, differentiating OSA patients with predominant anatomical factors from OSA patients with a combination of anatomical and non-anatomical factors [3].

The passive critical closing pressure (Pcrit) is the most important measurement of pharyngeal collapsibility, where a high Pcrit (>2 cmH_2_O) is related to high pharyngeal collapsibility [3].

Continuous Positive Airway Pressure (CPAP) is recognized as the first-line standard treatment for OSA, but long-term acceptance or adherence to CPAP is reported by the literature to be from 50 to 70% [4,5]. For this reason, multiple alternative treatment options are being advocated. Treatment options for OSA may include weight loss, mandibular advancement devices (MAD), positional therapy, and upper airways (UA) surgery, including hypoglossal nerve stimulation [6,7,8,9]. Furthermore, orofacial myofunctional therapy and drug therapy represent an emerging treatment option for OSA [10,11]. However, with expanded treatment options, the question arises of how to select the best treatment option, especially when considering UA surgery, knowing that the best outcomes are achievable with CPAP in OSA patients with the highest pharyngeal collapsibility (high Pcrit > −2 cmH_2_O), whereas less pharyngeal collapsibility (low Pcrit < −2 cmH_2_O) is related to more success with non-CPAP treatments [12].

UA surgery is often recommended for treating OSA patients who have refused or cannot tolerate CPAP, and the main result achievable by the surgery is UA expansion, and/or stabilization, and/or tissue removal to different UA levels [8]. The targeted approach to the surgical care of OSA is not a simple algorithm based on only clinical examination or UA evaluation findings. Multiple factors need to be considered, including the patient’s OSA physiologic endotype and clinical phenotype. Findings from each of these features must be incorporated into the best available clinical evidence and patient needs and preferences. These data then allow for a more targeted and customized approach [13].

This paper aims to analyze the data collected from medical history, sleep studies, and clinical examination, which could help an otorhinolaryngologist in detecting the preponderance of anatomical factors in UA collapsibility and its role in apnea events. These data may potentially influence the selection and the outcomes of a range of surgical procedures.

## 2. Materials and Methods

A comprehensive review of the English-language literature on UA surgery for OSA treatment was performed. A selection of the references was carried out in PubMed, EMBASE, Cochrane, and CENTRAL electronic database up to January 2021 using the following search keywords: “sleep apnea” OR “OSA” OR “OSAS” OR “phenotyping” OR “physiological Traits” combined with the use of the AND function with “ OSAS surgery” OR “palatal surgery” OR “uvulopharingopalatoplasty” OR “expansion sphincter-pharyngoplasty” OR “lateral pharyngoplasty” OR “relocation pharyngoplasty” OR “anterior palatoplasty” OR “hyoid suspension” OR ”maxillo-mandibular advancement” OR “hypoglossal nerve stimulation” OR “transoral robotic tongue reduction” OR “TORS’’ OR ‘‘barbed palatoplasty” to better select the research. To further reduce the risk of incomplete literature analysis, a manual search through the bibliography of the included papers was carried out.

## 3. Medical History

Outpatient setting offers a significant amount of clinical information that needs to be interpreted by an otorhinolaryngologist before asking for further diagnostic exams or opting for a specific therapeutic choice.

Collecting clinical history is certainly one of the main steps of a sleep ambulatory session. The goal is to identify the important traits to guide surgical decision making (see Table 1). For those patients who have not a comprehensive medical evaluation, the use of a tool such as the STOP-BANG questionnaire allows one to determine the risk for moderate to severe OSA and helps to decide on further diagnostic sleep tests [14,15].

## 4. Sleep Studies

As already mentioned, OSA is a quite complex pathophysiological disease characterized by physiological traits (endotypes), represented by high pharyngeal collapsibility (Pcrit), neuromuscular activity impairment, high LG, and low AT, which predict the clinical expression of OSA (phenotypes) [3,16]. The main endotypes are difficult to derive in daily practice and are limited to research sleep studies and/or via mathematical algorithms [16]. Otherwise, sleep studies and clinical examination allow an estimation of pharyngeal collapsibility and the other PTs.

Genta et al. reported higher Pcrit values (>−2.5 cmH_2_O) in OSA patients with a preponderance of obstructive apneas compared with OSA patients with a preponderance of hypopneas (A/H ratio > 1) and observed a linear relationship between BMI and Pcrit [12].

Landry et al. retrospectively demonstrated that a CPAP value ≤8 cmH_2_O identifies an OSA patient with low Pcrit (<−2 cmH_2_O), who could be selected for a non-CPAP treatment option, UA surgery included [17,18].

Edwards et al. showed that at least two of three of the following parameters identify OSA patients with a low arousal threshold (sensitivity of 80.4%; specificity of 88.0%): Apnea Hypopnea Index (AHI) < 30 events per hour, nadir oxygen > 82%, A/H ratio < 58.3% [19].

Finally, a high number of central or mixed apneas and polysomnographic patterns such as Cheyne Stokes breathing may identify OSA patients with high LG [20].

Hence, before considering UA surgery for OSA patients, it is essential to analyze the outcome of a polysomnographic (PSG) examination. The full-night, in-laboratory, attended-PSG (type 1) is referred to as the gold standard for the diagnosis of OSA, but it is a time-consuming and expensive examination, not always feasible in all suspicious SDB patients [21,22].

The American Academy of Sleep Medicine (AASM) has provided a guideline in which a type 1, 2, or 3 sleep study is recommended as pivotal in the diagnostic work-up to assess the severity of OSA and the position dependency, and differentiate between obstructive or central events [21,22].

The home sleep apnea test (HSAT, type 3) was introduced in the diagnostic assessment of patients with a high likelihood of OSA, and practice parameters for its application and evaluation have been reported in the literature [23].

Several types of polysomnographic data, which can be identified on an HSAT, can suggest the presence and grade of anatomical and non-anatomical pathophysiological factors:

- Pharyngeal collapsibility is higher when apnea events are predominant in comparison with hypopnea events (A/H ratio > 1), and clinical examination has excluded UA anatomical obstruction, such as palatine tonsil hypertrophy, base of the tongue hypertrophy, and so on [12].
- The analysis of flow limitation curves allows one to identify the potential site of pharyngeal collapse [24]
- AHI of non-supine position less than 50% of that of the supine position identify positional OSA (POSA) [25].
- Even if it is not possible to identify HSAT with the Rapid Eye Movement (REM) phase, the detection of clusters of phasic desaturations every about 90 min will suggest the occurrence of the events during the REM phase (REM-related OSA patients) [26,27].

Finally, the otorhinolaryngologist must also pay attention to detect the presence of severe cardiorespiratory, neuromuscular, or cerebrovascular diseases and related drug treatment, or symptoms of other sleep disorder(s), which preclude an adequate interpretation of HSAT data, and therefore a full-night PSG may be considered (see Table 2) [23].

## 5. Clinical Examination

General clinical examination, UA evaluation through flexible awake and drug-induced endoscopy (DISE), and imaging techniques such as lateral cephalograms or facial/neck CT scans allow physicians to recognize some typical anatomic patterns, which are strongly related to UA collapsibility due to preponderant anatomical factors.

The most important of the general features to analyze remain age and Body Mass Index (BMI), due to the strong correlation between age (range: 50–70 years), BMI > 30, and severity of OSA [28].

Several literature data deal with the relation between UA clinical examination and UA anatomical collapsibility [3,13,20,21,29]. Many of these, however, poorly predict surgical outcomes in practice. Consequently, different classification of the anatomical sub-sites of the UA has been proposed and used to identify patients at high risk of OSA. The clinical examination that correlates best with surgical outcomes is the Friedman staging system [30].

Friedman tongue position evaluates the visibility of velopharyngeal structures according to the position of the tongue:

- the uvula and tonsils/pillar (Friedman Tongue Position: FTP I),
- most of the uvula but not the tonsils/pillar (FTP IIa),
- the entire soft palate to the uvular base (FTP IIb),
- some of the soft palate with the distal end absent (FTP III)
- only the hard palate (FTP IV).

Concerning the palatine tonsil size, the author suggests the following grading system:

- absence of tonsillar tissue (grade 0)
- within the pillars (grade 1)
- extended to the pillars (grade 2)
- extended past the pillars (grade 3)
- extended to the midline (grade 4).

To correlate the main patient’s anatomical finding with the phenotypes of OSA, Friedman et al. introduced a surgical staging system, which considers the relation between tongue volume, palatine tonsil size, and patient BMI (see Table 3).

According to the Friedman staging and grading systems, an OSA patient with low FTP and grade 3–4 palatine tonsils hypertrophy represents the best candidate for the palatal surgery, with a success rate greater than 80% (stage I disease), even if they have a severe AHI at the sleep study. Otherwise, OSA patients with high FTP and low palatine tonsils hypertrophy (stage III disease) are not favorable candidates for palatal surgery alone, with a low success rate of 8% (see Table 3). Treatment options for stage III OSA patients could be multilevel surgery. In Friedman stage I-III OSA patients, a high UA collapsibility (high Pcrit) is quite probable, but the identification of the site/sites of obstruction is of pivotal importance for the surgical outcomes. According to Friedman’s suggestions, OSA patients with BMI over 40 and/or with significant craniofacial or other anatomic abnormalities (stage IV disease) are not candidates for surgical treatment [30].

## 6. UA Endoscopic Examination

The UA endoscopic assessment in OSA patients represents an essential step in the clinical examination for staged UA surgery approaches. The awake UA endoscopic evaluation assesses the features of the nose, oropharynx, hypopharynx, and larynx. It is important to realize that structures of the UA anatomically overlap and physiologically interact.

The presence of obstructive anatomical abnormality of nasal cavities, such as nasal septal deviation, inferior turbinate hypertrophy, obstructive congestion of the nasal mucosa, sinus and nasal polyposis, and so on, is strongly related to the onset of snoring, representing the facilitating factors for the pharyngeal anatomical collapsibility and apneas [31].

The endoscopic observation of the retro-palatal area from the nasopharynx allows one to analyze the anatomical conformation of the soft palate. Below the inferior edge of the soft palate, it could be possible to examine the anatomy of the pharyngeal walls, the impact of palatine tonsils hypertrophy, and the hypopharyngeal and laryngeal area, mainly the anatomy and impact of the base of the tongue and the epiglottis conformation.

Fiber optic endoscopy is essential to evaluate the base of the tongue, one of the most important sites of hypopharyngeal collapse. Recently, Friedman et al. [32] have proposed the evaluation of the base of the tongue according to the extension of its lymphatic tissue hypertrophy (LTH), suggesting the following grading system:

- no lymphoid tissue (LTH0)
- scattered lymphoid tissue (LTH1)
- lymphoid tissue covering the entire tongue base, limited vertical thickness (LTH2)
- lymphoid tissue covering the entire tongue base, the significant vertical thickness of approximately 5–10 mm (LTH3)
- lymphoid tissue covering the entire tongue base, rising to or above the tip of the epiglottis, approximately 1 cm in height (LTH 4)

The Muller’s maneuver, although strongly related to the patient’s ability in performing an effective inspiration with mouth and nose closed, enables the assessment of the pharyngeal collapsibility, and could suggest the different pharyngeal patterns of obstruction. However, Mueller’s maneuver is carried out in an awake patient, and the presence pharyngeal muscular tonicity is the main limitation of the procedure [33].

DISE, when performed in the standardized protocol [34,35,36,37], can offer additional information concerning the pharyngeal collapsibility and patterns of obstruction, allowing the choice of the best surgical procedure.

Woodson et al. suggested that a DISE assessment of multiple pharyngeal anatomical landmarks (hard palate, anatomic genu of the soft palate, uvula muscle in the velum, and lateral walls at the velopharynx) could suggest the pattern of the pharyngeal lumen at the retropalatal region and its importance when understanding postoperative surgical failure [38].

The soft palate anatomic genu (alpha angle) is defined by the tensor veli palatini length and its relationship with palatal aponeurosis.

An obtuse alpha angle identifies a larger airway size at the retropalatal area (distance between the hard palate and posterior pharyngeal wall). In this case, the shape of the collapse pattern is circular and, consequently, more favorable to palatal surgery. Conversely, an acute alpha angle is related to an anteroposterior collapse pattern, which is less favorable to palatal surgery [38,39].

Considering that the author reported the oblique pattern (obtuse alpha angle) as the most common pattern in his OSA patients’ series, a DISE assessment of retropalatal collapse is important for the decision of the specific palatal surgical procedure [38].

Furthermore, Vanderveken et al. reported that the DISE palatal pattern of anteroposterior collapse is related to a high success rate for hypoglossal nerve stimulation surgery. Conversely, a DISE palatal concentric collapse represents a negative predictive factor for hypoglossal nerve stimulation surgery success rate [40].

Moreover, DISE allows an accurate evaluation of lateral pharyngeal wall collapse and the magnitude of primary or secondary epiglottis collapse [34].

Finally, apart from grades 3–4 of LTH according to Friedman’s grading classification, an accurate evaluation of lingual tonsil pattern of collapse could be realized only under sedation [32].

## 7. UA Imaging Examination

Imaging examination can lead clinicians towards a specific diagnostic framing. Cephalometric measurements on lateral teleradiography include several anatomical landmarks, such as the posterior airway space, UA length, and hyoid-mandibular plane perpendicular distance [41,42,43].

Furthermore, literature data reported that the UA length, measured as the vertical distance between the superior-posterior hyoid and the inferior-posterior hard palate, parallel to the long axis of the airway, was associated with the presence and severity of OSA and pharyngeal collapsibility. Therefore, UA length seems to have a high sensitivity and a high specificity for predicting the presence of OSA [41,42,43].

The role of cephalometry in predicting treatment outcomes still needs to be ascertained. However, a review by Choi et al. highlights how a low position of the hyoid bone (long hyoid-mandibular plane perpendicular distance) is a negative predictor for the success of palatal surgery, thus suggesting the role of lateral oropharyngeal walls and tongue in the genesis of a multilevel collapse in this specific pattern of OSA patients [44].

CT scans play a role in OSA patients with maxillofacial syndromic abnormalities and are usually performed as a protocol study for maxillofacial surgery [45].

Finally, head and neck MRI is useful for a detailed study of the hypopharyngeal area, mainly for OSA candidates for surgical lingual tonsil reduction [46].

## 8. Oropharyngeal Surgery

Palatal surgery represents the first UA surgery applied as a treatment option for OSA [47,48]. Over the years, the most-performed surgical procedure for OSA has been uvulopalatopharyngoplasty (UPPP) with or without tonsillectomy (T). UPPP ± T aims to increase the retropalatal lumen and reduce the collapsibility of the pharynx, by resection of the free edge of the uvula and soft palate, often in combination with a tonsillectomy. However, this procedure was associated with a high incidence of unfavorable postoperative complications and morbidities [49]. In the short and long term, the most typical problems reported in these patients were dysphagia, rhinolalia, velopharyngeal insufficiency, nasopharyngeal regurgitation, soft palatal edema, and abnormal scarring with velopharyngeal stenosis [50]. Besides, the tissue resection required for these techniques caused a thick fibrotic scar on the palatal edge that, touching and abrading the base of the tongue, resulted in throat discomfort or foreign body sensation in the throat. Finally, a systematic review and meta-analysis published by Stuck et al. assessed the effects of UPPP in adult OSA patients, performed as a single surgical procedure, demonstrating an extremely broad improvement of AHI, and success/response rates ranged from 35 to 95.2% in different studies [47].

In recent years, lateral pharyngeal wall collapse has been reconsidered in OSA pathogenesis, and palatal surgical techniques have evolved from ablative techniques towards the remodeling of the pharyngeal lateral walls and enlargement of the velopharyngeal lumen [51].

The first of these techniques was the lateral pharyngoplasty, described by Cahali, which consists of a suture between superior pharyngeal muscle, previously sectioned in a craniocaudal direction, and the palatoglossus muscle [52]. In 2007, Pang and Woodson introduced the expansion sphincter pharyngoplasty (ESP), where the superior pharyngeal muscle remains intact, but the palatopharyngeus muscle is caudally sectioned, isolated, and sutured anterolaterally to the hamulus to expand the retropalatal area [53]. A recent systemic review and meta-analysis confirmed excellent results of this surgery for OSA treatment. The authors showed the mean pre-operative AHI improved from 40.0 ± 12.6 to 8.3 ± 5.2. The overall pro-rated pooled success rate for all the patients was 86.3% [54].

Of the different lateral pharyngoplasty techniques used, barbed reposition pharyngoplasty (BRP) has recently become a promising palatal surgery technique [55]. BRP has been described in a pilot study by Vicini et al. in 2015 as a quick, easy, and effective technique for treating a velopharyngeal obstruction in OSA patients. The authors proposed the use of barbed suture (knotless bidirectional resorbable suture) for obtaining a suspension of the palatopharyngeal muscle at the pterygomandibular raphe and expansion of the lateral walls of the oropharynx without tissue resection of the soft palate region [55]. The effectiveness of BRP was confirmed by several prospective multicentric and randomized studies [56,57,58]. Furthermore, preliminary results of a retrospective comparison study between UPPP, ESP, and BRP showed that BRP and ESP can be considered an effective procedure based on the postoperative outcomes. Both techniques proved to be superior to UPPP in postoperative complications and reduction in the number of apneas/hypopneas [59].

## 9. Hypopharyngeal Surgery

The base of the tongue represents the main site of hypopharyngeal collapse during apnea events [60,61]. Primary or secondary epiglottic as well as lateral hypopharyngeal wall collapse could be associated with tongue obstruction phenomena [62].

The surgical options available for hypopharyngeal apneas include tongue reduction procedures, such as midline glossectomy, lingualplasty, lingual tonsillectomy, and radiofrequency reduction, or tongue advancement/stabilization procedures, such as genioglossus advancement, hyoid suspension, and tongue suspension [8].

In 1999, Chabolle et al. described open midline glossectomy with hyoid epiglottoplasty [63]. Suh et al. reported a significant improvement in AHI in 56% of patients undergoing midline glossectomy in a cohort of 50 patients. The best results were obtained in patients with Friedman tongue position 3. Dysphagia, significant odynophagia, dysphonia, and aspiration are the most frequent complications of this kind of surgical procedure and must be emphasized during counseling with patient [64].

In the last years, trans-oral robotic surgery (TORS) has gained a central role as a tongue reduction surgical procedure. Meccariello et al. performed a review and meta-analysis of the published articles concerning TORS for OSA, including for the analysis 8/195 studies, which involved a total of 820 cases. The mean age was 49 ± 3.27 and 285 patients underwent a previous sleep apnea surgery. Conventional UPPP was the most common palatal procedure, combined with TORS. In all but one article, the success rate was defined as a 50% reduction of pre-operative AHI and an AHI < 20. Pre-operative and post-operative AHI means were, respectively, 46.88 and 20.24 with a mean rate of failure of 36.1% (*n* = 365). Transient dysphagia represented the most common complication (7.2%), followed by bleeding (4.2%) [61].

Recently, a review by Cammaroto et al. showed the benefits and drawbacks of TORS and coblation tongue base surgeries. For these authors, the use of the same surgical techniques performed with TORS or coblation allows one to reach similar outcomes. However, the authors concluded that the application of robotic technology, despite its high costs, might give slightly better results, allowing a wider surgical view and more consistent removal of lingual tissue [65].

Maxillomandibular advancement (MMA) is considered the most successful OSA surgical procedure, with a success rate of 86%, even if meta-analyses were performed in a limited patient population [66]. The MMA success rate is generally related to the UA expansion achieved by the surgery, but its effect on the upper pharynx is more incisive than its effect on the hypopharynx [67]. One of the most important findings endoscopically described in postoperative MMA patients is the improvement in lateral wall stiffness [68].

Hypoglossal nerve stimulation (HNS) is probably the most recent innovation in OSA treatment. HNS exploits neurostimulation to decrease UA collapsibility, allowing a multilevel UA improvement in a single surgery. At this moment, the strongest contraindication to HNS is the complete concentric collapse of the soft palate detected during DISE, which is an essential assessment tool for pre-operative patient selection [40,69].

Three different systems are now available in the market: The Inspire Medical System^®^ (Inc., Maple Grove, MN, USA), the ImThera^®^ system, and the new Genio™ system (Nyxoah SA, Mont-Saint-Guibert, Belgium). These systems stimulate the hypoglossal nerve differently over diverse portions of the nerve, thus leading to a different muscular activation. However, all these systems have shown comparable effectiveness, adverse effect incidence, adherence to treatment, and patient satisfaction [70].

## 10. Discussion

Currently, there are no worldwide standardized methods or algorithms for the selection of OSA patients suitable for UA surgery, and several new surgical techniques and modifications have been introduced in the last 40 years.

To date, UA surgical long-term outcomes evaluation are still limited by several factors: different UA surgical procedures have been introduced, associated with several modifications of the same original techniques, frequently applied in a patient population of a limited number.

Generally, the surgical decision is related to the surgeon’s preference, experience, and local technology available. Moreover, there are no worldwide accepted surgical protocols for the OSA patient’s different anatomical sites.

The UA surgical outcomes reported are generally analyzed considering only the Apnea/Hypopnea Index (AHI) improvement, without including other parameters, such as daytime sleepiness and quality of life.

The identification of non-anatomical traits (LG and AT) is not routinely verified, and the OSA patient selection is often based only on the anatomical characteristics, identified by different methods. Consequently, the surgical outcomes reported are variable and unpredictable, and strongly related to the presence of unfavorable LG or AT.

Three papers have focused attention on the relation between UA surgery and pharyngeal collapsibility, showing that the main impact of UA surgery is on the modification of passive collapsibility [71,72,73].

Schwartz et al. measured the Pcrit before and after UPPP in 13 OSA patients and observed a significant reduction of post-operative Pcrit (0.2 ± 2.4 to −3.1 ± 5.4 cm cmH_2_O (*p* = 0.016). The definition of response was post-surgical NREM AHI reduction >50% in comparison to the pre-surgical AHI. No difference in pre-operative Pcrit was observed between responders and non-responders, whereas significant differences in post-operative Pcrit were reported only in the responders group, in which a significant fall in Pcrit from −0.8 ±3.0 to −7.3 ± −4.9 cmH_2_O (*p* = 0.01) was highlighted. Taking into consideration that the soft palate is the main target of UPPP, it is reasonable to conclude that the responders group consisted mainly of patients with an isolated site of collapse at the palatal level [71].

Woodson et al. compared transpalatal advancement pharyngoplasty with UPPP, focusing on UA characteristics changes: UA cross-sectional size and collapsibility were measured after UPPP and transpalatal advancement pharyngoplasty. UA characteristics and Pcrit were measured following each procedure in seven OSA patients. Transpalatal advancement pharyngoplasty lead to an increase of maximal retropalatal airway size (measured using a quantitative endoscopic technique) of 220% (from 61.5 to 135.0 mm^2^; *p* < 0.001) and a reduction of Pcrit of 9.2 cmH_2_O (from 5 to −4.2 cmH_2_O; *p* < 0.001). These outcomes supported transpalatal advancement pharyngoplasty as a more effective technique for the improvement of retropalatal size and collapsibility in comparison with conventional UPPP [72].

Furthermore, Woodson et al. compared transpalatal advancement pharyngoplasty with UPPP in another series including six OSA patients. Transpalatal advancement pharyngoplasty was more effective than UPPP at increasing maximal retropalatal airway size (measured using a quantitative endoscopic technique) by 321%, and decreased Pcrit from 4.7 ± 1.6 to −3.8 ± 0.7 cmH_2_O (*p* < 0.01). Respiratory disturbance index (RDI) decreased from 74.5 ± 13.5 to 29.2 ± 9 events/hour postoperatively (*p* < 0.05). Moreover, the transpalatal advancement pharyngoplasty increased the pharyngeal area by 120% (*p* = 0.001), and Pcrit decreased by 9.2 cmH_2_O (*p* < 0.01). Finally, the maximal anteroposterior length (MAX-AP) and maximal lateral radius were increased by 90% (*p* = 0.01) and 60% (*p* < 0.001), respectively, and the Pcrit changed in relation to airway size (r^2^ = 0.44, *p* < 0.05) [73].

Summarizing, Woodson’s research confirmed that the primary effect of palatal surgery is the improvement of the anatomical pharyngeal collapse, which is strictly related to the grade of reduction of Pcrit (up to more than 9 cmH_2_O). These outcomes allow an understanding of the significant role of UA surgery in the treatment of OSA, underlining its potential effectiveness, even in selected patients with severe pharyngeal anatomical collapsibility [72,73].

Analyzing UA surgical outcomes, it has been frequently speculated that the response to surgery is also variable because it does not improve the non-anatomical factors (LG and AT), which often are unfavorable in non-responders, but it is also possible that UA surgery can modify non-anatomical PTs as well.

Li et al. evaluated Chinese subjects, hypothesizing that high LG could decrease after UA surgery, contributing to the reduction in sleep apnea severity. The authors compared a control group (15 participants) to 30 OSA patients who underwent UA surgery (surgery group). UA surgery improved the AHI; 15/30 (50%) of OSA patients were responders (≥50% reduction in AHI and post-surgery AHI < 20 events/h), 8/30 (26.7%) were cured (postoperative AHI < 10 events/h), and the mean pre/post AHI changed from 60.8 to 18.4 events/h (*p* < 0.001). In the surgery group, preoperative and postoperative LG decreased from 0.70 (0.58–0.80) to 0.53 (0.46–0.63), respectively (*p* < 0.001). LG was not modified in the control group. There was a positive association between the decrease in LG and the improvement of AHI (*p* = 0.025). The authors concluded that LG was reduced by UA surgical treatment, and this reduction suggests that high LG may be, at least partially, acquired in OSA [74].

Joosten et al. analyzed the effect of UA surgery on extrapharyngeal PTs and tried to test the value of LG and AT in predicting surgical success rate. Forty-six OSA patients underwent UA surgery, consisting mainly in septoplasty associated or not with inferior turbinoplasty, ESP associated or not with tonsillectomy, lingual tonsil surgery, and tongue channeling. In summary 39/46 patients underwent multilevel UA surgery (20/46 tongue surgery, 4/46 only tonsillectomy, 3/46 only nasal surgery). The overall surgical treatment responders reported were 26% (AHI 50% reduction and a post-operative AHI < 10 events/h). Surgery decreased the AHI (39.1 ± 4.2 vs. 26.5 ± 3.6 events/hour; *p* < 0.005) but did not modify LG (0.45 ± 0.08 vs. 0.45 ± 0.12; *p* = 0.278), both in the whole group and in the two subgroups of responders and non-responders. AT decreased both in the whole group and in the subgroup of responders, demonstrating that the AT is at least in part an acquired PT and the improvement of OSA improves AT. Surgical responders had a lower baseline LG (0.38 ± 0.02 vs. 0.48 ± 0.01, *p* < 0.05) and lower LG was a significant predictor of surgical success, after controlling for covariates (logistic regression *p* = 0.018) [75].

Another important factor is the patient’s age, considering that in elderly patients, the degree of collapsibility of the retropalatal structures is significantly greater than in younger patients [76]. Moreover, the genioglossal responsiveness to negative intrapharyngeal pressure appears to deteriorate with age. These anatomical and functional characteristics could lead to a higher degree of failure in terms of the functional outcome of the various surgical pharyngoplasty techniques. Furthermore, surgical complications could be greater in the elderly than in young patients. In this context, in their recent observational study, Gouveia et al. analyzed the complications of surgery in 107 OSA patients aged > 65 years, showing that elderly patients undergoing sleep surgery have an increased risk of postoperative complications compared to younger people treated with the same procedures [77]. In particular, the elderly group had higher rates of wound dehiscence, postoperative bleeding, and postoperative urinary tract infections. The specific complication rate for the elderly patients reported was 7.5%, and multivariate analysis identified age >65 as an independent risk factor for perioperative complications [77].

Considering all the above concepts, it is possible to identify the best candidate for UA surgery by the analysis of medical history, clinical features, and basic examination tests (see Table 4).

## 11. Conclusions

Currently, the therapeutic armamentarium for OSA comprises several treatment options. To provide effective treatment for OSA, careful consideration of the individual patient, available medical and surgical therapies, and inherent risks and complications of those interventions must be considered. A growing body of evidence is becoming available, supporting the practice of other treatment modalities, especially mandibular advancement devices (MADs), weight loss, positional therapy (PT), and UA surgery. The approach to treating OSA is steadily moving from a CPAP-centered “one-size-fits-all” approach to individualized multimodality treatment of UA obstruction during sleep.

Patients with high Pcrit (high collapsibility) respond best to CPAP, positional therapy, and weight loss, with an adjunctive or salvage role for surgical interventions and oral appliances. Patients with intermediate increases in Pcrit (mild–moderate collapsibility) may also have concomitant altered LG, neuromuscular impairment, or AT. Therefore, these patients may benefit from anatomical or non-anatomical treatments. Patients with negligible airway collapse, with only marginally increased Pcrit, have OSA for non-anatomical reasons, and may be treated with pharmacologic manipulation of LG or AT.

Without changing the bony confines of the facial skeleton, we can change the paradigm of surgery from altering UA not to simply decrease the obstructions or enlarge the UA size but to stiffen the lateral wall to minimize the dynamic collapse. In our opinion, this could play an important role in the evolution of OSA surgery through the next years.

## Figures and Tables

**Table 1:** Obstructive Sleep Apnea: signs, symptoms, and complications.

Signs and Symptoms	Complications
Loud snoring Episodes in which you stop breathing during sleep—which would be reported by another person Gasping for air during sleep Awakening with a dry mouth Morning headache Difficulty staying asleep (insomnia) Excessive daytime sleepiness (hypersomnia) measured with Epworth questionnaire (ESS) Difficulty paying attention while awake Irritability Erectile dysfunction Nocturia	Daytime fatigue. Asthma Atrial fibrillation Heart and blood vessels diseases, such as atherosclerosis, heart attacks, heart failure, difficult-to-control high blood pressure, and stroke Cognitive and behavioral disorders, such as decreases in attention, vigilance, concentration, motor skills, and verbal and visuo-spatial memory, as well as dementia in older adults. In children, sleep apnea has been associated with learning disabilities Metabolic disorders, including glucose intolerance and type 2 diabetes Complications with medications and surgery Liver problems Sleep-deprived partners

**Table 2:** Systemic diseases for full-night PSG indication.

1. Significant cardiorespiratory disease 2. History of stroke 3. Severe insomnia 4. Potential respiratory muscle weakness due to neuromuscular condition 5. Awake hypoventilation or high risk of sleep-related hypoventilation 6. Symptoms of other significant sleep disorder(s) 7. Chronic opioid medication use

**Table 3:** Friedman staging system as determined by Friedman tongue position (FTP), tonsil size, and BMI.

STAGE	FTP	TONSIL SIZE	BMI
I	I, IIa, IIb	3 or 4	<40
II	I, IIa, IIb III or IV	0, 1, or 2 3 or 4	<40 <40
III	III or IV	0, 1, or 2	<40
IV *	I–IV	0–4	>40

*: all patients with significant craniofacial or other anatomic abnormalities.

**Table 4:** Ideal/non-ideal candidates for UA surgery by the analysis of medical history, clinical features, and basic examination tests.

	Ideal Candidates for UA Surgery	Unfavorable Candidates for UA Surgery
**CLINICAL FINDINGS**	- Age < 65 years old - BMI < 35	- Age > 65 years old - BMI > 35 - Systemic comorbidity with high anesthestic risk - Use of muscle relaxant drugs
**ANATOMICAL FINDINGS**	- Mallampati score 1–3 - Friedman stage I and II - FTP III and IV (consider TORS) - Isolated circular collapse of the velopharyngeal region and obtuse alpha angle (DISE evaluation) - Primary epiglottis collapse (DISE evaluation)	- Mallampati score 4 - Friedman stage III and IV - Low hyoid position and a more verticalization of the base of the tongue - Lateral hypopharyngeal collapse (DISE evaluation) - Craniofacial anomalies/microretrognathia (consider mandibular advancement) MMA)
**POLYSOMNOGRAPHIC/CPAP FINDINGS**	- A/H ratio < 1 - CPAP pressure values less than 8 cm cmH_2_O (low Pcrit) - Flow limitation analysis predicts potential site of pharyngeal collapse	- A/H ratio > 1 - CPAP pressure values greater than 8 cm cmH_2_O (high Pcrit) - Prolonged obstructive events and low oxygen nadirs (high AT) - High central or mixed apneas score (high LG) - Cheyne Stokes Breathing (high LG)
